# Achieving Universal Access for Human Immunodeficiency Virus and Tuberculosis: Potential Prevention Impact of an Integrated Multi-Disease Prevention Campaign in Kenya

**DOI:** 10.1155/2012/412643

**Published:** 2012-05-07

**Authors:** Reuben Granich, Nicolas Muraguri, Alexandre Doyen, Navneet Garg, Brian G. Williams

**Affiliations:** ^1^Treatment and Care Unit, Department of HIV/AIDS, World Health Organization, Avenue Appia 20, 1211 Geneva, Switzerland; ^2^Ministry of Public Health and Sanitation, Government of Kenya, Nairobi, Kenya; ^3^Vestergaard Frandsen (EA) Ltd., Waiyaki Way, ABC Place, P.O. Box 66889, Nairobi 00800, Kenya; ^4^Innovation Centre, Vestergaard Frandsen, Chemin de Messidor 5-7, 1006 Lausanne, Switzerland; ^5^South African Centre for Epidemiological Modeling and Analysis, Stellenbosch, South Africa

## Abstract

In 2009, Government of Kenya with key stakeholders implemented an integrated multi-disease prevention campaign for water-borne diseases, malaria and HIV in Kisii District, Nyanza Province. The three day campaign, targeting 5000 people, included testing and counseling (HTC), condoms, long-lasting insecticide-treated bednets, and water filters. People with HIV were offered on-site CD4 cell counts, condoms, co-trimoxazole, and HIV clinic referral. We analysed the CD4 distributions from a district hospital cohort, campaign participants and from the 2007 Kenya Aids Indicator Survey (KAIS). Of the 5198 individuals participating in the campaign, all received HTC, 329 (6.3%) tested positive, and 255 (5%) were newly diagnosed (median CD4 cell count 536 cells/*μ*L). The hospital cohort and KAIS results included 1,284 initial CD4 counts (median 348/L) and 306 initial CD4 counts (median 550/*μ*L), respectively (campaign and KAIS CD4 distributions *P* = 0.346; hospital cohort distribution was lower *P* < 0.001 and *P* < 0.001). A Nyanza Province campaign strategy including ART <350 CD4 cell count could avert approximately 35,000 HIV infections and 1,240 TB cases annually. Community-based integrated public health campaigns could be a potential solution to reach universal access and Millennium Development Goals.

## 1. Introduction

 In 30 years since the start of the human immunodeficiency virus (HIV) pandemic over 25 million people have died [1, 2]. In 2010, an estimated 34 million people were living with HIV and 67% of them lived in sub-Saharan Africa [3].   Antiretroviral therapy (ART) has considerable potential to save lives while reducing the HIV transmission [4–7]. By the end of 2010, 6.6 million people were on antiretroviral treatment (ART) in the world [3]. Despite this remarkable achievement, an estimated 7.5 million people with CD4 cell counts <350/*μ*L were still in need of treatment [3]. Without a dramatic reduction in HIV incidence it is unlikely that we will be able to meet the growing demand for ART [3, 8]. Addressing this prevention gap will require innovative approaches to improving access to HIV services including HIV testing and counselling (HTC) and ART.

 Community-based efforts, including outreach beyond health facilities, may provide one approach to help bridge this gap. Of the 34 million people living with HIV, a majority are still unaware of their HIV status [3]. WHO, recognizing the need to markedly scale-up access to HTC, has recommended provider initiated HIV testing and counselling [9]. The Kenya National HIV and AIDS Strategic Plan III includes an HTC target of 18 million (80%) of people 15–49 years of age to be newly tested by 2013; however, despite increases in facility-based HTC the 2007 Kenya Aids Indicator Survey (KAIS) found that only 36% of adults have ever had an HIV test, and less than 20% of HIV-infected adults know that they are infected [10, 11]. Obstacles to access to HTC include a shortage of trained counsellors, limited services, high transportation costs, limited test kit availability, and stigma [10–15]. Home-based HTC offers an important potential strategy to reach targets by expanding access beyond health care facilities. With a reported uptake of up to 90% in some settings, home-based HTC also provides an opportunity for couples counselling and mutual disclosure [15]. In Uganda's Bushenyi District, a 2.5 year multi-disease house-to-house HIV testing and counselling programme tested 264,996 (94% acceptance) people in 92,984 (63%) households [14].

 Although home-based service delivery is feasible in many settings, it can be time and labour-intensive. Complementary community-based health campaigns are well suited to delivering services to the rural poor and have been used to deliver HIV counselling and testing and other simple interventions to large populations [16, 17]. By combining multiple interventions and placing a larger proportion of the transaction costs onto the provider, integrated multi-disease campaigns create efficiencies for both the consumer and provider [16–19]. There is considerable experience with the health campaign approach including the mass distribution of insecticide-treated bednets which have quickly reached high coverage levels at low cost and are associated with declines in child and adult mortality in east Africa [16–19]. More recent work in sub-Saharan Africa has focused on bundling multiple interventions into a multi-disease prevention package which includes long-lasting impregnated bednets, water purification systems, preventive health education, condoms, and cotrimoxazole prophylaxis for HIV-infected adults [16, 17, 20–22].

 Building on the previous community-based campaign experience in Kenya's Kakamega District, in 2009 we implemented a similar multi-disease prevention (MDP) campaign in Kisii District, Nyanza Province [16]. We applied lessons learned to pilot streamlined HTC protocols, provide same-day, onsite access to CD4 cell counts, and strengthen linkage to care. Our study examines the HIV component of the Kisii District campaign and compares CD4 count distributions to explore whether a multi-disease campaign strategy can improve earlier access to HIV diagnosis and treatment. We also project the potential HIV and TB prevention impact achieved by reaching people earlier for different ART eligibility scenarios.

## 2. Methods

### 2.1. Multi-Disease Prevention Campaign

 In 2008 the Ministry of Health Kenya, the United States Government Centers for Disease Control Kenya, Community Housing Foundation (a local NGO), and Vestergaard Frandsen (a private sector manufacturing company focused on products that address the MDGs) implemented a 7 day multi-disease prevention campaign that reached 47,311 (92%) of adults 15–49 years old in Kakamega District, Western Province of Kenya. The campaign was in line with the Kenyan National AIDS Strategic Plan III's target to reach 80% of 15–64 year olds and provided interventions to address HIV, malaria, and diarrhea [10, 16]. Point-of-care CD4 counts were also piloted in selected sites [16]. Nyanza Province in western Kenya, has a high incidence of malaria, diarrhoeal disease, and tuberculosis [3, 10, 11, 23–25]. In 2007, an estimated 15% of 15–64 year olds were living with HIV [11] which has contributed to the high annual TB incidence (353 per 100,000 population) [11, 24, 25]. Standard operating procedures from the Kakamega campaign [16] were modified for the Kisii District Campaign as described below.

**Figure 1:**
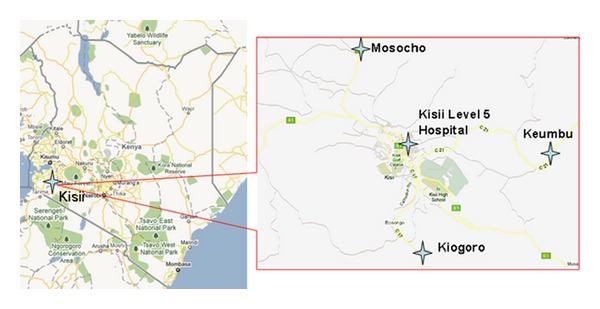
Map of Kenya showing Kisii, Nyanza Province, and inset showing the location of the three campaign sites and Kisii Level 5 Hospital (Kisii Town).

 In September 2009 we implemented a three-day multi-disease prevention campaign that targeted diarrhoeal diseases, malaria and HIV in Kisii District, Nyanza Province (population 4,392,000). Three peri-urban campaign sites were set up around health facilities in the periphery of Kisii (Figure 1 map). The campaign was designed to (1) apply lessons learned from previous campaigns to a peri-urban setting, (2) pilot improved HIV testing and counselling protocols, (3) strengthen the referral system for improved linkage to care, (4) determine the feasibility of providing same day CD4 cell count testing for all HIV-positive participants and, (5) explore the potential impact of campaigns for early identification for HIV prevention and care services, and (6) examine differences between campaign, hospital, and provincial populations.

 The campaign was planned to provide services for 5000 adults within 3 days by using a specific protocol adapted to mass campaign settings. We conducted the campaign over a weekend (Saturday, Sunday, and Monday) to ensure maximum participation of both men and women and limit disruptions to routine services. A pre-campaign social mobilization exercise started one month before the start date and engaged the community using village “baraza” forums with local chiefs, radio and print messaging, and town cries with mobile trucks. Participants were informed about campaign services, campaign sites and provided health education messages around diarrhea, malaria, HIV, and STDs. They were offered services on a first-come first-serve basis and census lists from 2008, identification cards, and indelible finger print dye were used to ensure that participants could only participate once in the campaign (all participants received finger dye irrespective of the services accessed or serostatus). To ensure that people accessing services were from the immediate local area, we used village elders and government officers working at the village level to advertise the campaign and carefully recorded location information during the registration process.

 A total of 90 counselors were hired and trained to use the mass testing protocol designed for community-based campaigns with a target of 25 clients per day. Pretest counselling was offered by trained Health Communication Officers to groups of 20 participants selected for age and gender as they waited to meet the HTC counsellors. Confidentiality, consent, and counselling were assured by issuance of cards with a unique identifier number during the registration process and it was emphasized to all participants that HTC was entirely voluntary and that everyone would receive the other interventions whether or not they opted for HIV testing. HTC was provided on an “opt-in” basis and written consent was required for testing. Quality of HTC was insured by the use of certified counsellors, refresher training courses, a supervision system which employed one supervisor for every 10 counsellors, sending 1 out of 50 blood samples for reconfirmation with a different diagnostic method (PCR), and exit interviews by trained staff for all campaign participants. The exit interviews were used to assure quality and to improve services on a real-time basis.

 Participants who tested HIV positive received a 3-month supply of cotrimoxazole, same-day on-site CD4 count measurement, psychosocial support, local referral for further care, and were offered enrolment in a support network by peer counsellors. Linkage to care was given a high priority and planned for through various interventions. Counselors emphasized the importance of care during post-test counselling. Members from local people living with HIV (PLWHA) support networks were enrolled and trained for the implementation of the PLWHA *navigator* approach. As part of the *navigator* strategy, people testing HIV positive were offered further counselling by assigned PLWHA counsellors and, with consent, were enrolled into local support groups. Most participants opted to allow follow-up visits and provided name, address, unique identifier number, and phone number. PLWHA counsellors, using a list of clients, checked in with health centers on a monthly basis and, if necessary, made follow-up household visits [26].

 The Kisii campaign included provision of one long-lasting insecticide-treated net per participant, water filters (individual filter for men, household filter for women), 60 condoms per person, and health education encompassing HIV, sexually transmitted infections, malaria, and water-borne diseases. The unit cost per person by disease was $6.27 for malaria (nets and training), $15.80 for diarrhea (filters and training), and $9.91 for HIV (test kits, counselling, condoms, and CD4 testing) [16, 27]. Using logistic and expenditure data from the 2008 Lurambi District Campaign, [16] the cost of a scaled-up replication (SUR) was estimated assuming reliance on local managers, potential efficiencies of scale, and other adjustments (Jim Kahn, personal communication). The SUR cost of $31.98 per person included 67% for commodities (mainly water filters and bed nets) and 20% for personnel.

### 2.2. Measurement and Analysis of CD4 Cell Count Distributions

 Absolute CD4 cell counts and total lymphocyte counts were performed by portable Guava AUTOCD4 flow cytometers manufactured by Millipore. All three sites were equipped with a unit with a single 150 amperes battery (7 hour off-grid capacity). Samples were processed in batches and had a 45-minute incubation time and 4-minute processing time. Each unit had a trained machine operator and a trained nurse or other health care provider responsible for drawing 10 *μ*L of whole blood (EDTA) and preparing samples for analysis. As patients waited for their results, they were given additional psychosocial counselling by a counsellor living with HIV. For external quality control, 5 percent of all blood samples were sent for confirmation at Kisii Level 5 Hospital Laboratory using a Becton Dickinson Facs Calibur Flow Cytometer. The Kisii Hospital laboratory routinely sends 10% of blood samples to CDC Kisumu for external quality control.

 To create a matching historical cohort and a baseline for comparison, we abstracted the medical records, including the first measured CD4 count, for all newly diagnosed patients aged 15 and above from March to August 2009 at the HIV/AIDS Patient Support Center in the Kisi District Level 5 Hospital (apex of district health care facilities). All CD4 measurements for this cohort were made using the same Kisii Level 5 Hospital laboratory Becton Dickinson Facs Calibur Flow Cytometer.

 We analyzed the CD4 data from the Kisii campaign, the reference hospital and Nyanza province data from the recently performed KAIS survey [11]. The 2007 KAIS is the first national population-based survey of Kenya that obtained representative estimates on behavioral, clinical and biologic indicators for HIV/AIDS. The 2007 KAIS was conducted among a sample of households selected from all eight provinces in the country, covering both rural and urban areas (more detailed methods are described in detail elsewhere) [11].

### 2.3. Projecting the Potential HIV and TB Prevention Impact of Early Detection

 For our comparison and projections we used data from those who were reported as being newly diagnosed—all were assumed to be ART naive. Cumulative distribution functions were compared using the standard Kolmogorov-Smirnov test. We estimated the potential benefits of early identification and starting ART for three scenarios (1) <250 CD4 cells (status quo), (2) <350 CD4 cells, and (3) immediate ART irrespective of CD4 cell count.

**Figure 2:**
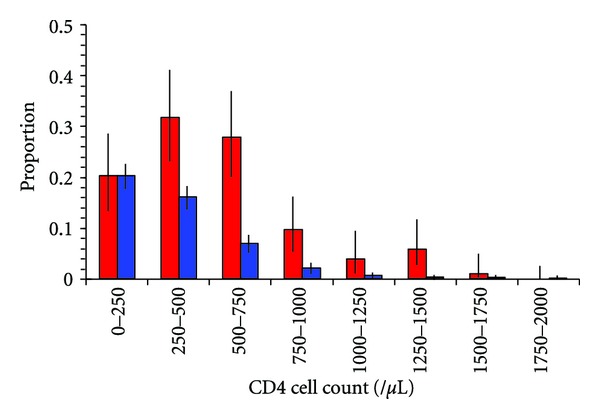
Comparison of the CD4 cell count distribution in Nyanza Province (red; KAIS survey) and the Kisii Hospital cohort (blue). The data for the hospital cohort are scaled to match the KAIS data for the lowest CD4 cell count range and the differences in the heights of the bars for the higher ranges show the proportion that are missed in the hospital cohort.

 To estimate the proportion of people with CD4 cell counts that are missed under passive clinic-based case-finding but would be found using a campaign approach, we assumed that everyone with a CD4 cell count below 250/*μ*L will present to a health facility before they die. We scaled the proportions in the “hospital reference” data so as to match the proportion in the KAIS data set below 250/*μ*L (Figure 2). Applying the scaling proportion allows us to see the differences in proportions of people that are missing in the hospital cohort at the higher CD4 levels. This in turn enables us to obtain an approximate estimate of the increase in the number of people who would be put onto ART and the number of TB cases that would be averted by adopting a campaign approach.

 The population of Nyanza province is 4.4 million of whom 2.9 million are adults with around 435,000 (15%) who are estimated to be HIV positive [11]. We estimated the number of HIV-positive TB patients in Nyanza Province in two ways. First, the case-notification rate of HIV-positive TB patients in Nyanza is 189 per 100,000 population giving about 8,314 HIV/TB cases per year [25]. Second, the life-time risk of TB for those not receiving ART has been estimated to be 13% [28]. With a mean life expectancy of HIV-positive people of ten years, this means that the annual risk of TB-disease is 1.3% and we expect there to be about 435,000 × 0.013 = 5655 case of TB in HIV-positive people in the province each year. Taking the average of these two estimates, the expected number is 6,985 per year. ART reduces the incidence of TB by about 71% [28], so that if everyone started ART immediately they were found to be HIV-positive the number of TB cases averted would be 4,959. This enables us to estimate the reduction in the number of TB cases that we expect each year under the campaign and passive clinic-based case-finding approaches.

 To estimate the number of new HIV transmissions averted, we assumed that the epidemic is in a steady state so that each person with HIV infects one other person before they die. Assuming that the CD4 cell count is 750/*μ*L immediately after seroconversion [29] we multiply the number started on ART by the proportion of time for which they are on ART (the CD4 cell count at the time at diagnosis in the different scenarios is used to calculate the amount of time on and off ART). To derive the HIV infections averted we compared the projected outcome using these assumptions with “no ART.” To simplify the analysis, we did not include the additional prevention benefits of the long-term reduction in TB transmission and treatment of identified TB cases. We also did not factor in WHO recommended IPT or infection control for TB which is not yet in widespread use in Kenya [30].

The study protocol was reviewed by the Kenya Ministry of Public Health and Sanitation and considered to be part of on-going program monitoring and evaluation. The study represented a private-public partnership and funding for the study was provided by the Kenya Ministry of Public Health as part of routine public health services. Ministry of Health Kenya and Vestergaard Frandsen funded the campaign; MOH provided campaign personnel, HIV test kits, and condoms. The decision to conduct, analyse, and submit the study was taken by the Ministry of Health and WHO.

## 3. Results

### 3.1. Multi-Disease Prevention Campaign

 Over a three-day period, the campaign reached 5198 individuals aged over 15 years with a 100% uptake of the HIV counselling and testing and multi-disease preventive package. Counselors worked 8 hour days starting from 8:00 AM and tested around 25 clients per day (100% of target). Clients who were found to be HIV negative were provided HTC in about 20 minutes, while those who were diagnosed with HIV were given HTC counseling in about 38 minutes. The process from drawing blood to getting CD4 cell count results usually took around two hours (mean 119; range 47–191 minutes).

 Of the 5198, 2090 (40%) were males. Of the 329 participants who tested HIV positive, 71 (22%) were males; HIV prevalence among males was 3.3% and 8.3% for females. This difference of HIV prevalence between genders reflects the 2010 antenatal care sentinel surveillance results of 8.7% among women in Kisii District sites [31]. A separate study that included a subsample of the people from the Kisii campaign and others evaluated factors affecting linkage to care and found that 81% of people who consented to follow-up visited the referral clinic by 10 months after the campaign [26].

### 3.2. Analysis of CD4 Cell Count Distributions

 Of the 258 (4.9%) who were newly diagnosed with HIV (71 knew their status before campaign), CD4 count determination was performed for 255 (98%). The median CD4 count was 536 cells/*μ*L (IQR 348 to 760;) with 13% having a CD4 count <250 cell/*μ*L and 25% a CD4 cell count <350 cells/*μ*L (Table 1).

 Of the 1284 patients in the Kisii Hospital reference cohort, 350 (27%) were male (age range 15–61; CD4 count range 1–1862) and 934 (73%) were female (age range 15–69; CD4 count range 1–2560). The first CD4 counts from the 1284 patients had a median of 348 (IQR 185 to 551) with 34% having a CD4 count <250 cell/*μ*L and 50% a CD4 cell count <350 cells/*μ*L (Table 1).

 The results obtained from the 2007 KAIS data base for Nyanza Province included 1585 females, 1386 (87%) tested, 240 (17%) HIV positive, 218 (91%) not on ART, and 203 (85%) with CD4 counts. Of the 1229 males surveyed, 994 (81%) were tested, 123 (12%) HIV positive, 108 (88%) not on ART, and 103 (95%) with CD4 counts. The median CD4 count overall was 550 cells/*μ*L (IQR 305 to 785). Table 1 shows that the CD4 cell count data from the campaign for Kisii are not significantly different from the KAIS data for Nyanza (*P* = 0.346).

### 3.3. Projecting HIV and TB Prevention Impact of Early Detection

Figure 2 shows that the Hospital reference cohort has significantly lower median CD4 cell counts when compared with the Campaign and KAIS data.  Table 2 shows that using our scaled estimation approach with either campaign or hospital-based strategies, current ≤250 ART eligibility criteria results in around 38,000 people started on ART and about 645 cases of TB will be averted in Nyanza Province. Increasing the CD4 cell count eligibility to ≤350 combined with the passive case-finding approach increases the number starting ART to 56,000, averts 26,000 new HIV infections, and prevents 942 TB cases. However, the ≤350 ART eligibility criteria combined with the campaign approach would translate into an estimated 74,000 people starting ART, thereby averting 35,000 new HIV infections and preventing 1,240 TB cases per year. Starting at a CD4 cell count of 500/*μ*L gives an even greater relative advantage to the campaign approach with 129,000, or 2.6 times as many people started on ART, and 2,182 total or 2.6 times as many TB cases averted using the campaign approach when compared with the passive case-finding approach.

## 4. Discussion

This three-day integrated multi-disease prevention campaign reached over five thousand people in Kisii district including over 200 people who were unaware that they were living with HIV. The uptake of HCT in the campaign is comparable to the high rates of over 90% observed in home-based, door-to-door testing interventions implemented in Uganda [15, 32, 33] and Kenya [34] and was achieved in considerably less time. Similar to previous campaigns, [16] successful implementation of this campaign may have been due to the engagement of the community leadership, delivery of services at convenient locations near participants' homes and the multi-disease prevention approach which included concomitant distribution of free nets, water filters, and condoms. Although access to laboratory tests including CD4 levels has been a major barrier to expanding access to ART [35, 36], the campaign successfully delivered same-day CD4 level testing results for all of the newly identified people with HIV.

 Delayed diagnosis and access to ART have significant public health implications for both the individual and the community. Expanded access to HCT linked with point-of-care CD4 testing has considerable potential to support the implementation of WHO's recommendation to start ART for everyone with a CD4 ≤ 350/*μ*L [37]. Comparison of CD4 counts from campaign participants with the hospital cohort CD4 data and the recent national survey suggests that the campaign identified people significantly earlier in the course of their HIV disease. This makes intuitive sense as it reaches people before they are symptomatic and is supported by other studies examining the use of community-based services outside health facilities [38]. Although we do not present the data, the 80% linkage to care for people diagnosed with HIV in this campaign at 10 months was better than in many other settings [39] but required setting up a robust follow-up system. The data also suggest that increasing the threshold to 350/*μ*L combined with the standard passive facility-based case-finding approach could increase the number of people in Nyanza who need to start ART by a factor of 1.9 or 56,000 people. However, the campaign approach combined with optimal linkage to care could increase the number receiving ART by a factor of 3.7 or 74,000 additional people—an additional 18,000 people who were unaware of their HIV status and who were eligible but not on ART. Our simple projections using a stable generalized epidemic setting suggest that an active campaign approach to identify those with CD4 cell count <350 could prevent 10,000 HIV transmissions, 76,000 deaths and 3600 TB cases per year. Although more complex projections for the province and country are beyond the scope of this paper, improving access to early ART through a campaign approach could have significant public health and economic benefits including preventing morbidity, mortality, disease transmission, and reducing costs to the individual, health system, and society [4, 6, 40].

Short intense multi-disease campaigns face a number of challenges including maintaining efficiency and quality of service provision and linkage to care while dealing with large numbers of people. Previous work in Kenya and elsewhere suggests that careful consensus building and micro-planning with community leaders and key health care providers is required to mobilize resources and provide high-quality services for the temporary surge of participants in the campaign [16]. The various TB and HIV prevention scenarios modeled would only be achievable under conditions of a high linkage to care after the campaign which requires postcampaign systems monitoring health care facility attendance, active followup, and local support networks. Another significant challenge is the cost of the campaign. Preliminary analyses suggest that despite the relative high costs per person [27] the campaign is likely to be cost effective in part due to the multi-disease approach and the numbers of people reached in a short period of time. Arguably, delivering health care services from fixed facilities is also costly and often does not reach stated objectives.

There are important limitations to our study. The comparison of the hospital, province, and campaign CD4 data may have been influenced by a number of biases introduced from the selection of the three populations. Specifically, it is difficult to say with certainty that the three subpopulations that we compared are similar given the different ways that people accessed the hospital, campaign and KAIS survey (e.g., nonresponse, refusal, and missing CD4 counts). Additionally, there are potential confounders that may have affected the CD4 results including the difference in methods to determine CD4 counts, diurnal rhythms, physical and psychological stress, pregnancy, drug administration, tuberculosis, and viral infections. However, the similarity of the campaign data with the provincial data for Nyanza is reassuring and the lower CD4 counts of those who are ill and seeking care in a hospital setting make sense. Our assumption that people coming into the hospital for care were not referred from a peripheral site and the high linkage to care may have resulted in optimistic projections favoring the campaign strategy. However, despite our lack of certainty regarding the projected benefits which relied on crude estimates, we are likely to be directionally correct and a more sophisticated modeling approach may provide additional insights.

We are far from achieving universal access and there is increasing interest in new approaches to ensuring early and equitable access to ART and other HIV services. This multi-disease prevention campaign presents an operational proof of concept for the expanded access to HTC and same-day CD4 testing that is required for many countries to reach national HIV and TB prevention goals. Multi-disease integrated campaigns have considerable potential and may represent an important conceptual breakthrough in our efforts to achieve national health objectives reflected in the Millennium Development Goals [41].

## Figures and Tables

**Table 1:** CD 4 values from the campaign, hospital reference, and KAIS data sets. The table gives *N*, the number of people for whom a CD4 cell count was done, the median CD4 cell count, and the proportion of those tested that are below 250, 350, and 500 cells/*μ*L. Numbers in brackets are percentages. Using a Kolmogorov-Smirnov test, The CD4 cell count distribution for the Hospital Reference data set is significantly different from the other two (*P* < 0.001 in both cases) but the Campaign and KAIS data sets are not significantly different (*P* = 0.346).

	Campaign	Hospital reference	Nyanza KAIS
*N*	255	1284	306

Median/*μ*L	536	348	550
*N* < 250	33 (13%)	436 (34%)	52 (17%)
*N* < 350	64 (25%)	642 (50%)	92 (30%)
*N* < 500	112 (44%)	899 (70%)	141 (46%)

*N* < 750	187 (74%)	1137 (89%)	220 (72%)

*N* < 1000	228 (90%)	1215 (95%)	258 (84%)

**Table 2:** Projected prevention impact of campaign approach by CD4 eligibility criteria for Nyanza Province.

	Campaign approach				Passive case-finding			
CD4 cell count at start of treatment (/*μ*L)	HIV-positive population started on ART (%)	Number started on ART (thousands)	HIV infections averted per year (thousands)	TB cases averted per year	HIV-positive population started on ART (%)	Number started on ART (thousands)	HIV infections averted per year (thousands)	TB cases averted per year
*≤*250	13	38	13	645	13	38	13	645
*≤*350	25	74	35	1240	19	56	26	942
*≤*500	44	129	86	2182	27	79	53	1339
Immediate	100	294	294	4959	38	112	112	1884
